# Comparative analysis of bypass vs. stent for coronary revascularization using an ex-vivo organ care system in an animal model

**DOI:** 10.1038/s41598-026-50599-8

**Published:** 2026-05-12

**Authors:** Philipp Lindenhahn, Rabea Hinkel, L. Christian Napp, Johanna Diekmann, Jens P. Bankstahl, Frank M. Bengel, Holger Volk, Bernhard Hiebl, Jan B. Hinrichs, Lena S. Becker, Jannik Richter, Iliyana Pepelanova, Danny Jonigk, Klaus Höffler, Axel Haverich, Tim Kaufeld

**Affiliations:** 1 Department of Cardiothoracic, Transplantation and Vascular Surgery, Hannover Medical School, Hannover, Germany; 2 Department of Small Animal Medicine and Surgery, University of Veterinary Medicine Hanover, Hannover, Germany; 3 Laboratory Animal Science Unit, German Primate Center, Leibniz Institute for Primate Research, Göttingen, Germany; 4 German Center for Cardiovascular Research (DZHK), Partner Site Lower Saxony, Göttingen, Germany; 5 Institute for Animal Hygiene, Animal Welfare and Farm Animal Behavior, University of Veterinary Medicine Hanover, Hannover, Germany; 6 Department of Cardiology and Angiology, Hannover Medical School, Hannover, Germany; 7 Department of Nuclear Medicine, Hannover Medical School, Hannover, Germany; 8 Department of Diagnostic and Interventional Radiology, Hannover Medical School, Institute for Diagnostic and Interventional Radiology, Hannover, Germany; 9 Institute of Technical Chemistry, Leibniz University of Hannover, Hannover, Germany; 10 Medical Faculty, Institute of Pathology, RWTH Aachen University, 52074 Aachen, Germany; 11 Hannover Medical School, Institute of Pathology, Hannover, Germany; 12 German Center for Lung Research (DZL), BREATH, Hannover, Germany; 13 Center for Integrated Oncology Aachen Bonn Cologne Düsseldorf (CIO ABCD), 52074 Aachen, Germany; 14 Department of Small Animal Medicine and Surgery, University of Veterinary Medicine Hannover, Bünteweg 2, Hannover, Germany

**Keywords:** Bypass, Stent, Atherosclerosis, Coronary artery disease, Cardiology, Diseases, Medical research

## Abstract

Many patients with coronary artery disease are eligible for either percutaneous coronary intervention (PCI) or coronary artery bypass grafting (CABG). Technical and clinical aspects influence the decision for either treatment. However, biological effects of PCI and CABG on long-term coronary artery anatomy and physiology should be considered but are largely unknown. Eight German landrace swine were used in this study. An artificial atherosclerotic plaque (AAP) was implanted into the left anterior descending coronary artery (LAD) to simulate coronary atherosclerosis. Physiological changes of PCI and surgical CABG in vascular and perivascular tissue were assessed in an ex vivo setting (organ care system OCS). Furthermore, radiological and nuclear imaging was performed using single photon emission computed tomography (SPECT) and computed tomography (CT). Furthermore, interventional (PCI) and surgical (CABG) treatment was evaluated using an ex-vivo setting. Lymphatic flow and myocardial perfusion were improved in pigs in the CABG group compared to the PCI group. The PCI group showed a significantly higher mean count number proximal to the intervention in the LAD area. Stenting experiments showed a significantly higher mean count number proximal to the intervention in the LAD area. This effect could also be demonstrated macroscopically, as myocardial infarct areas were smaller and myocardial function was better after defibrillation in the OCS (organ care system) in the CABG treated hearts. The artificial atherosclerotic plaque model in porcine hearts is a new valuable tool to simulate coronary artery stenosis without damaging other organs. It may serve as a tool for future medical testing and for further specific research on coronary artery physiology. Our data suggest that the cardiac lymphatic vascular system and perfusion capability are partly restricted after PCI as compared to CABG.

## Introduction

Coronary heart disease (CHD) is one of the most common causes of death worldwide^1–9^. An epidemiological study from 2016 shows that CHD is responsible for 20% of deaths in Europe^10^. The cardiovascular disease statistics 2021 show a prevalence of CHD in the United States of 20.1 million people (7.2%) between 2015 and 2018 (age > 20 years, both sexes) and a reported mortality of 360.900 in 2019 (all ages, both sexes)^11^.

The origin of atherosclerosis is still controversially discussed and can be caused under different circumstances. A build-up of fatty deposits (atheroma) on the walls of coronary arteries and a chronic inflammatory process of the diseased blood vessel is a characterized by narrowing of the affected blood vessel, potentially resulting in partially occlusion and culminating in a complete obstruction of the arterial lumen^12–14^. Suffering, of increased oxygen demands people first complain about angina pectoris based on an inadequate ejection of blood from the heart, irregular cardiac rhythms and myocardial infarctions. A clinical endpoint for the most tragic cases is the sudden cardiac death^15^. Also, there is increasing evidence that vasa vasorum (VV), the micro blood vessels in the adventitial and outer layer of the media of large blood vessels, play a central role in the pathogenesis of atherosclerosis. An endothelium dysfunction could cause a huge damage in microvessels and an obstruction of VV would peak into functional impairment followed by structural damage in the supplied vessel^16,17^.

PCI and CABG are beneath medication the interventional revascularization options to help these patients in need. Studies compare the two treatment methods and discuss CABG can prolong life in stable coronary artery disease^18–20^. Thus, PCI and CABG mechanisms may differ^21^.

Concrete figures from the German Heart Report 2021 show that stents were inserted in about 298.557 cases in 2020. Bypass surgeries were performed in 2020 (isolated and together with heart valve surgery) only in approximately 38,000 cases^22^. The indication and the choice of the procedure to be carried out have been controversially discussed for years, especially against the background of the sustainability of the treatment result. The influence of the lymphatic system has so far been ignored. There is no data describing the mechanical changes in the lymphatic vessels or perfusion of the coronary arteries caused by medical interventions via PCI, CABG and stent. Furthermore, it is still not clear why 20% of all patients who undergo coronary intervention need surgery within the following four years. In bypass-operated patients, this proportion is only 6%^22^. Compared to percutaneous coronary intervention, bypass surgery has a higher invasiveness and requires longer convalescence.

Our project thus enters a new field of research with high scientific relevance.

As in most diseases atherosclerosis is not only restricted to humans, as it is prevalent in a wide array of animals - herbivores, omnivores, carnivores and birds^23,24^.

The objective of this study was to insert an AAP in the LAD in porcine hearts. Our group previously described the establishment of this AAP^25^. To enhance the outcomes of interventional procedures such as angioplasty and stenting, a broad understanding of the mechanics of diseased arteries is crucial^26^. Understanding of both lumen gain and vessel injury post-stenting has led to greater interest in modelling stent-plaque-artery interactions and surgical procedures^26–33^.

## Materials and methods

### Animals

The study was approved by the LAVES (Lower Saxony State Office for Consumer Protection and Food Safety) and was carried out in accordance with the German Animal Welfare Act and Directive 2010/63/EU of the European Parliament and of the Council of 22 September 2010 on the protection of animals used for scientific purposes. The authors confirm that the study is reported in accordance with ARRIVE guidelines.

German landrace swine were used in this study (n8; both sexes) mean weight of 74 kg. The animals were purchased from the Ferkelerzeugergemeinschaft Hannover-Land w.V. (Springe, Germany). Sample size was based on feasibility for an exploratory study. Pigs are preferred as a non-rodent species in translational, human heart research due to the similarity in physiology, function, and anatomy of the cardiovascular system^34–36^. The pig epicardial coronary artery distribution closely resembles that of humans, although with fewer collateral vessels^37^.

General anesthesia and endotracheal intubation were utilized, and arterial carotid access was obtained by cut down. Anticoagulation with heparin was achieved to maintain an activated clotting time (ACT) _250 sec. The LAD was selected for the implantation of the AAP in each animal, size measured with IVUS (mean intra luminal diameter of 2.5 mm) and the target vessel for all the following research. The optimal vessel location for AAP implantation was in the proximal portion of each LAD, trying to avoid the occlusion of visible side branches, imitating a primary coronary stenosis that requires intervention. Via catheter the AAP got implanted into the anterior interventricular branch (RIVA, also known as the LAD) of the left coronary artery. Animals were randomly assigned to either the PCI group (*n* = 4) or the CABG group (*n* = 4) prior to the procedure. Four pigs are treated while under anesthesia with a stent immediately after plaque implantation (stent group SG). The other 4 animals received bypass surgery (bypass group BG) right after AAP implantation. For both groups it is important to treat the narrowing right away after the plaque implantation so that an ischemia time is as short as possible. To guarantee fast reperfusion via in situ coronary bypass graft, the chest was opened prior the invention.

### Pre-OP preparation

In this acute experiment, all measurements, and interventions up to the removal of the heart took place under anesthesia without awakening. The animals are anesthetized intramuscularly (i.m.) with a single dose of azaperone (2 mg / kg) and ketamine (10–15 mg / kg). The venous access via a peripheral vein catheter (PVC) is via the right or left ear vein. Then propofol is applied (3–5 mg / kg) intravenously (i.v.) until the onset of intubation. Lidocaine spray (xylocaine 10%) is applied to the larynx and intubation is done with an endotracheal tube. The animal’s skin is freed from bristles and cleaned in the corresponding operating areas in the preparation room.

The anesthesia is maintained by continuous intravenous infusion of 2-4.4 mg / kg / h propofol 2% and fentanyl (5–10 µg / kg / h i.v.) via a syringe pump. If necessary, isoflurane (1.0-1.5 vol% in an oxygen/air mixture) was administered via mechanical ventilation to achieve and maintain the appropriate depth of anaesthesia. During the surgical procedure, the animals are artificially ventilated in intermittent positive pressure ventilation (IPPV) mode. The respiratory rate is set to 12–16 breaths per minute. And the tidal volume to 8–15 ml / kg. If necessary, the blood gases are checked (BGA: Hb, electrolytes, etc.). For this, a volume of 2 ml of blood is taken from the central access.

After local anesthesia with lidocaine (2%, 2–3 ml), the internal jugular vein is prepared and a central venous catheter (CVC) is inserted using the Seldinger technique (for the application of drugs and blood sampling for blood gas analysis). An arterial access (internal carotid artery) is also created using the Seldinger technique. The accesses are fixed by skin staples with non-absorbable sutures.

At the end of the procedure, a bolus of propofol (10–20 mg/kg i.v.) and fentanyl (20–50 µg/kg i.v.) is administered to induce a deep terminal anaesthesia. All blood is withdrawn before the heart is removed. The euthanasia process is completed by confirming the final circulatory arrest during the explantation of the heart.

### In vivo measurements

The first measurements were taken before implantation of the AAP and repeated after bypass/stent treatment before heart collection.

#### Left ventricular pressure-volume analysis

The ventricular pressure–volume (PV) analysis is the reference method for the study of cardiac mechanics and enables the state-of-the-art investigation of ventricular performance^38,39^. End-systolic and end-diastolic PV and help relationships (ESPVRs and EDPVRs) can be used to quantify pump function, contractility, pathophysiological changes (e.g. heart failure, for testing the intraluminal application of the AAP^40^. To evaluate the heart state before and after surgery we compared the measurement of the end-systolic and end-diastolic pressures and volumes, ejection fraction, stroke volume and stroke work.

#### Angiography

Angiography was carried out under general anesthesia. After systemic heparinization (400 IU/kg body weight; Heparin-Natrium-25000; Ratiopharm), the left carotid artery was used for arterial access. A nonionic contrast agent (Imeron 350^®^; Bracco-Byk Gulden, Konstanz, Germany) was used for angiography. The left main coronary artery was engaged using a 14 F GORE^®^ DrySeal Flex Introducer Sheath and a Vistabritetip^®^ guiding catheter. Subsequently, a regional application of contrast agent is carried out by means of retrograde application via the anterior heart vein.

#### Intravascular ultrasound (IVUS)

A standard IVUS catheter (Volcano^®^ Eagle Eye Platinum, RX Digital IVUS Catheter) was advanced over a floppy coronary wire (Cordis/ATW^®^ All track wire, Floppy) and Coronary Guide Wire (Galeo Pro^®^, Straight, F, Standard, 0.014 inch, 300 cm, 5pcs) and manually pulled back for imaging the AAP as well as the coronary artery distal and proximal to the AAP. IVUS was used to measure the vascular inner diameters in the region of the LAD. Stationary IVUS imaging and analysis were completed at 4 mm increments relative to the anatomical landmark for at least 4 cardiac cycles.

### Plaque manufacturing

In an interdisciplinary setting with the Institute of Technical Chemistry, Leibniz University of Hannover, we engineered a casting mold to create an atherosclerotic plaque with the dimensions to fit in a porcine coronary artery. We designed, produced, and tested different materials. Oscillatory rheology experiments took place along with long-term stability tests assessed by microscopic examination and weight monitoring. Its components consist of a gelatin matrix from porcine skin mimicking the fibrous, cellular content, mixed with the lipid component of cholesterol and phospholipids from soybean, hydroxyapatite and finely grained calcium carbonate. For the implantability in future in vivo setups we made a cytotoxicity assessment, inserted the plaque in resected pig hearts and performed diagnostic imaging to visualize the plaque in its final position^25^.

### Plaque-stent Implantation

**Fig. 1:**
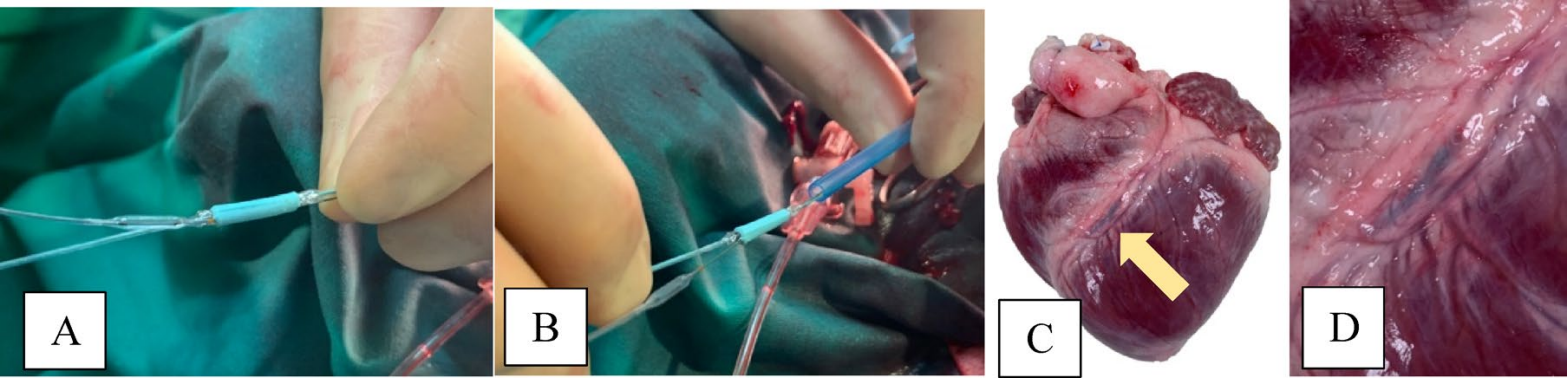
(**A**–**D**) Bare metal stent + AAP insertion through an introducer and a guiding catheter into the LAD.

Plaque implantation was performed under general anesthesia. After engaging the left main coronary artery with a 9 F guiding catheter, the AAP was mounted onto a commercial bare metal stent (CoCr Genous 2,75 × 18 mm) and fixed with 4 atmospheres inflation pressure (Fig. 1A,B). A standard floppy guide wire was placed into the peripheral LAD and the stent-AAP-construct was advanced to the LAD (Fig. 1C,D). After placement at the target zone (medial area of the LAD) the ballon was inflated at 20 atmospheres for 20 s to deploy the stent, leading to deployment of the AAP between the stent layer and the coronary artery intima. Implantation results was confirmed angiographically.

In the context of the surgical procedures, the process of plaque/stent implantation in conjunction with the in-vivo measurements and subsequent bypass anastomosis was a particular challenge. The experiment shows that the AAP can be advanced very well like a stent into the desired area of the LAD. Controlled x-raying using the C-Arm Unit is essential.

### Bypass

The bypass operation is carried out under anesthesia (see above) after the plaque has been placed (see above). For bypass surgery, local anesthesia is also used to eliminate pain (see 1.2.6). In a right-sided position, access to the heart is through a left-lateral thoracotomy in the 4th intercostal space. The lungs are carefully displaced with a wide pulmonary spatula over a damp abdominal cloth to provide better access. First, the left-sided mammary artery is prepared as a pedicle in its entire length. Side branches are closed by titanium clips and the artery is treated with papaverine. This is followed by the presentation and separation of the thymus fatty tissue as well as a longitudinal pericardiotomy. By placing and lightly tightening three pericardial sutures, a heart rotation to the right takes place for a sufficient representation of the RIVA. By applying a stabilizer, the movements of the heart are reduced without critically affecting the ejection performance of the heart.

The RIVA is bypassed with a thread and freed from epicardial adipose tissue. The artery is opened and a 1.5 mm shunt is placed. This is followed by the end-to-site anastomosis of the in situ mammary sinister artery graft with the RIVA in continuous suture technique on the beating heart using a 7.0 Prolene suture in off-pump coronary artery bypass (OPCAB) technique. After removal of the coronary shunt, the circulation of the two arteries occurs.

The ischemia time between APP implantation and therapeutic bypass/stenting was about 20 s in the experiments.

### Post operative measurements

At the end of the experiment, a bolus of propofol (10–20 mg/kg i.v.) and fentanyl (20–50 µg/kg i.v.) is administered to induce a deep terminal anaesthesia. The animal is then exsanguinated via total blood withdrawal. For the heart explantation, PERFADEX^®^ cardioplegia is administered in accordance with the Organ Care System (OCS) protocol to achieve cardiac arrest. The euthanasia process is completed by the final circulatory arrest confirmed during the removal of the heart.

After explantation, the heart was connected to the OCS and placed in the perfusion module with the anterior aspect facing upward. Perfusion of the heart was initiated with a pump flow of 900–1200 mL/min, with the aim of achieving the target coronary flow. The heart was perfused using standard OCS mode, maintaining mean aortic pressures of 60–80 mm Hg and coronary flows of 650–750 mL/min during the procedure. The heart was made to beat again and examined using single-photon emission tomography (SPECT) and computed tomography (CT). In addition, samples were taken for histological examination.

#### Organ care system

**Fig. 2:**
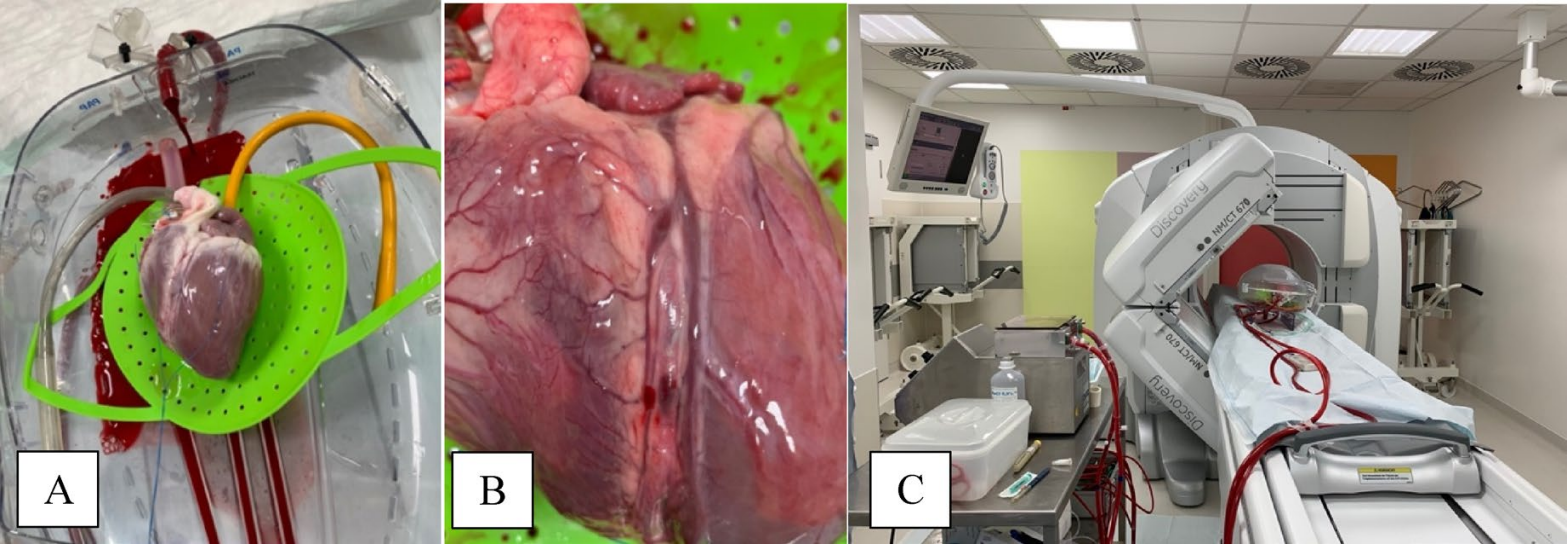
(**A**) porcine heart in the OCS facing with the anterior side upwards, (**B**) closer look to the LAD, (**C**) porcine heart within the OCS imaged with a SPECT/CT camera.

The Transmedics^®^ Organ Care System (OCS) utilizes an extracorporeal circuit to preserve the donor heart in a near-physiological, beating state by continuously perfusing it with warm, oxygenated blood (Fig. 2). Prior to explantation, approximately 1500 mL of donor blood is collected to prime the system. Following a brief period of cardioplegic arrest for retrieval, the heart is instrumented and mounted into the OCS heart module. Perfusion is typically established at a flow rate of 1 L/min, though this is adjusted based on specific graft parameters such as size or hypertrophy. The preservation solution—consisting of donor blood supplemented with nutrients, epinephrine, and a maintenance solution—is delivered into the ascending aorta to perfuse the coronary arteries and sustain the myocardium^41^.

Venous return from the coronary sinus collects in the right atrium. Since the venae cavae are occluded during this ex-vivo perfusion, the right ventricle ejects blood into the pulmonary artery. A cannula placed in the pulmonary artery returns this deoxygenated blood to the circuit, allowing for precise measurement of coronary flow, a key monitoring parameter^42^. Lactate levels are monitored via arterial and venous blood sampling to assess metabolic status and perfusion adequacy. Based on these data, aortic pressure and pump flow are regulated to ensure optimal preservation^41^. For this specific study, the OCS setup was modified to include a specialized organ chamber, an active drainage system, and extended perfusion lines to facilitate high-quality imaging (Fig. 3).

Following explantation of the heart, it was defibrillated using a Hellige device and maintained in a beating state through continuous stimulation via an external pacemaker (Medtronic). Physiological parameters, including blood gases, electrolytes, and lactate levels, were closely monitored using the Abbott I-STAT 1 analyzer. Electrolyte imbalances were corrected as needed, with targeted adjustments of potassium, calcium, and glucose to maintain homeostasis. For myocardial protection during explantation, PERFADEX^®^ 1.500 ml cardioplegia was used to achieve cardiac arrest and preserve tissue viability.

#### Nuclear medicine

**Fig. 3:**
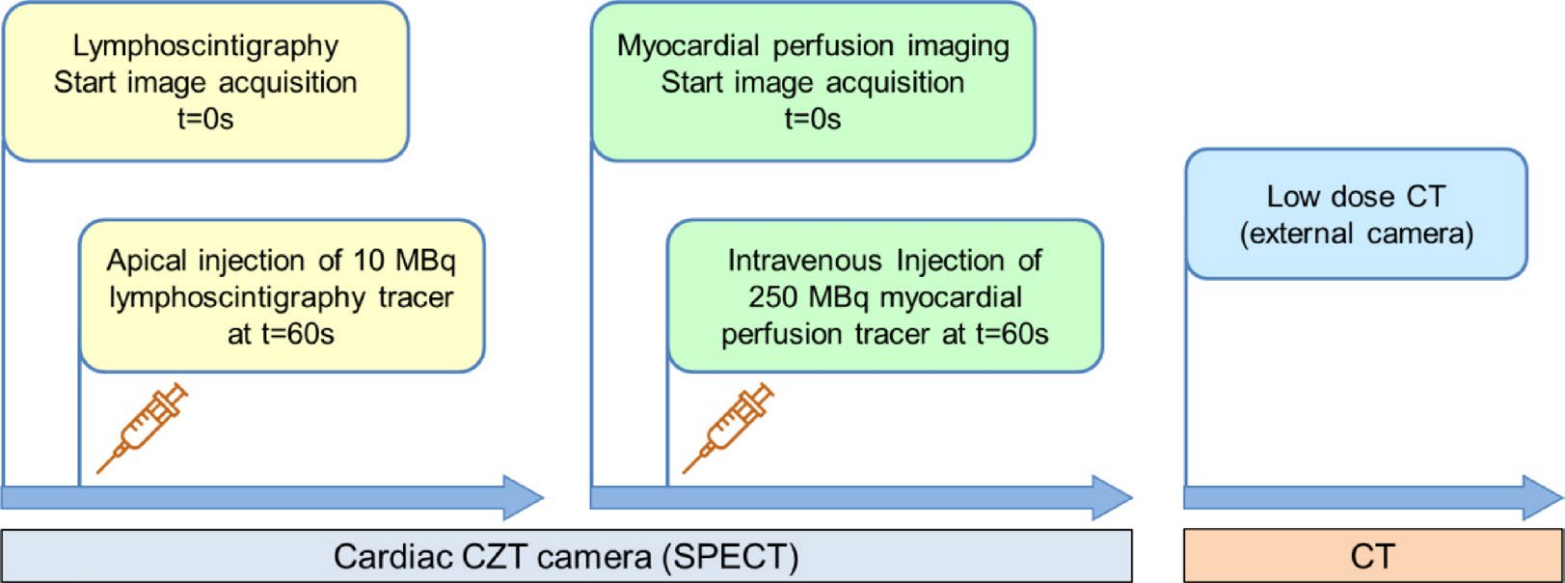
Imaging protocol - Single photon emission computed tomography (SPECT) and computed tomography (CT) imaging protocol for lymphoscintigraphy (yellow) and myocardial perfusion imaging (green).

##### Cardiac single photon emission computed tomography (SPECT) lymphoscintigraphy

After explantation, pig hearts with attached organ care system were visually centered in the field of view of a dedicated cardiac SPECT camera (Discovery NM 530c; GE Healthcare, Haifa, Israel) without prior tracer injection. List-mode image acquisition over 11 min was started to detect cardiac lymph transport. After 60 s, 10 MBq Nanocoll (GE Healthcare, consisting of ^99m^Tc–labeled human albumin nanoparticle in 0.2 mL saline) were injected directly into the apex of the left ventricular myocardium. Image reconstruction was performed with the camera-specific software (Xeleris, 4DM, GE Healthcare). Visual image analysis was conducted using commercial software (syngo.via; V50B, Siemens Healthcare). For additional semiquantitative analysis of lymph transport in the three coronary territories volumes of interest (VOIs) were placed in each coronary territory in the LV myocardium and mean counts were measured. The regional counts in the different coronary territories were then expressed as percentages of the total LV and statistically compared.

##### Myocardial perfusion imaging (MPI)

Directly after completed image acquisition of the lymphatic flow myocardial perfusion imaging was started without moving the heart in the field of view of the SPECT camera. Pig hearts were examined using a perfusion radiotracer (^99m^Tc-teboroxime, 250 MBq), which was injected intravenously and accumulates in cardiomyocytes. Myocardial perfusion SPECT was acquired over 11 min (Discovery NM 530c; GE Healthcare, Haifa, Israel). Image reconstruction and analysis see 3.7.2.1.

##### Computed tomography (CT)

An additive low-dose CT was conducted in all pig hearts directly after myocardial perfusion imaging for improved anatomical orientation on a different camera (GE D670; GE Healthcare, Haifa, Israel; camera specifications: 120 kV, 10 mA, 5 mm slices, matrix 512 × 512). Lymphoscintigraphy and myocardial perfusion images were fused with the CT for hybrid imaging (syngo.via; V50B, Siemens Healthcare).

#### Histopathology

Histopathological examination of the cardial tissue was performed at the Institute of Pathology, Hannover Medical School. To this end, representative tissue samples of the left anterior descending arteries of both groups were acquired and specimens were fixated with formaldehyde solution (4%) and paraffin embedded (FFPE). The LaserLabSolution Rowiak Group cut approx. 4 μm thick sections of the paraffin embedded heart tissue from both groups and stained them with hematoxylin/eosin-elastica (HE-Elastica) and masson goldner´s trichrome staining (MG). For further evaluation of ischemic, lymphatic / blood vessel and peripheral nerval injuries the stained slides were histopathologically assessed by light microscopy in a compartment-specific manner^43^. No blinding was performed.

## Results

### Measurements

#### Angiography/IVUS

**Fig. 4:**
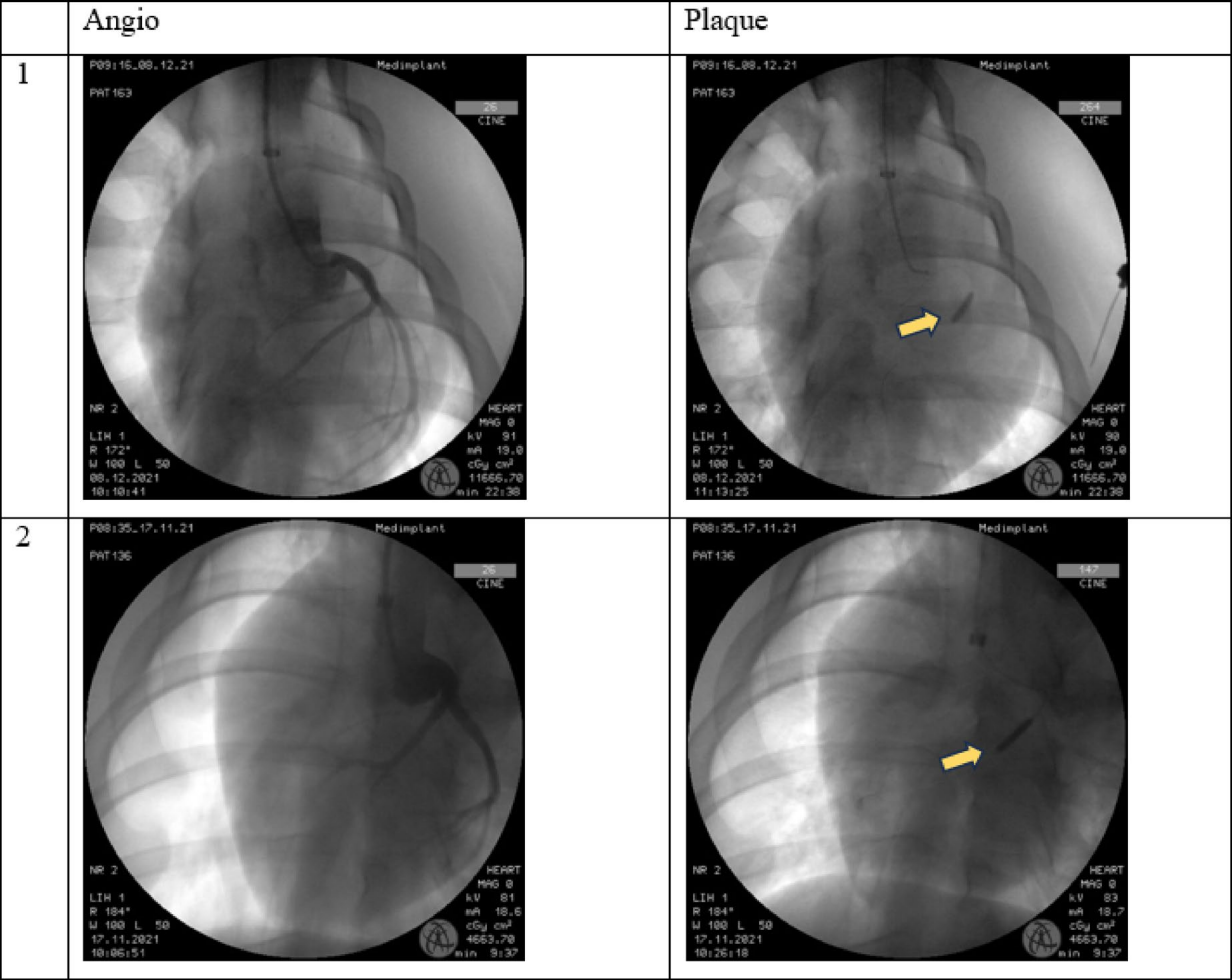
Angiography of the left anterior descending artery during AAP insertion. (**A**) LAD before stent deployment. (**B**) LAD after AAP deployment; yellow arrow: AAP.

AAP application resulted in a focal 50% stenosis of the LAD (average inner diameter of the LAD ∼2.7 mm). There was no evidence of myocardial hypoperfusion after stent deployment or bypass surgery. Retrograde infusion of contrast agent into the left coronary artery flowed into the native coronary vessels of the myocardium in each animal (Fig. 4).

**Fig. 5:**
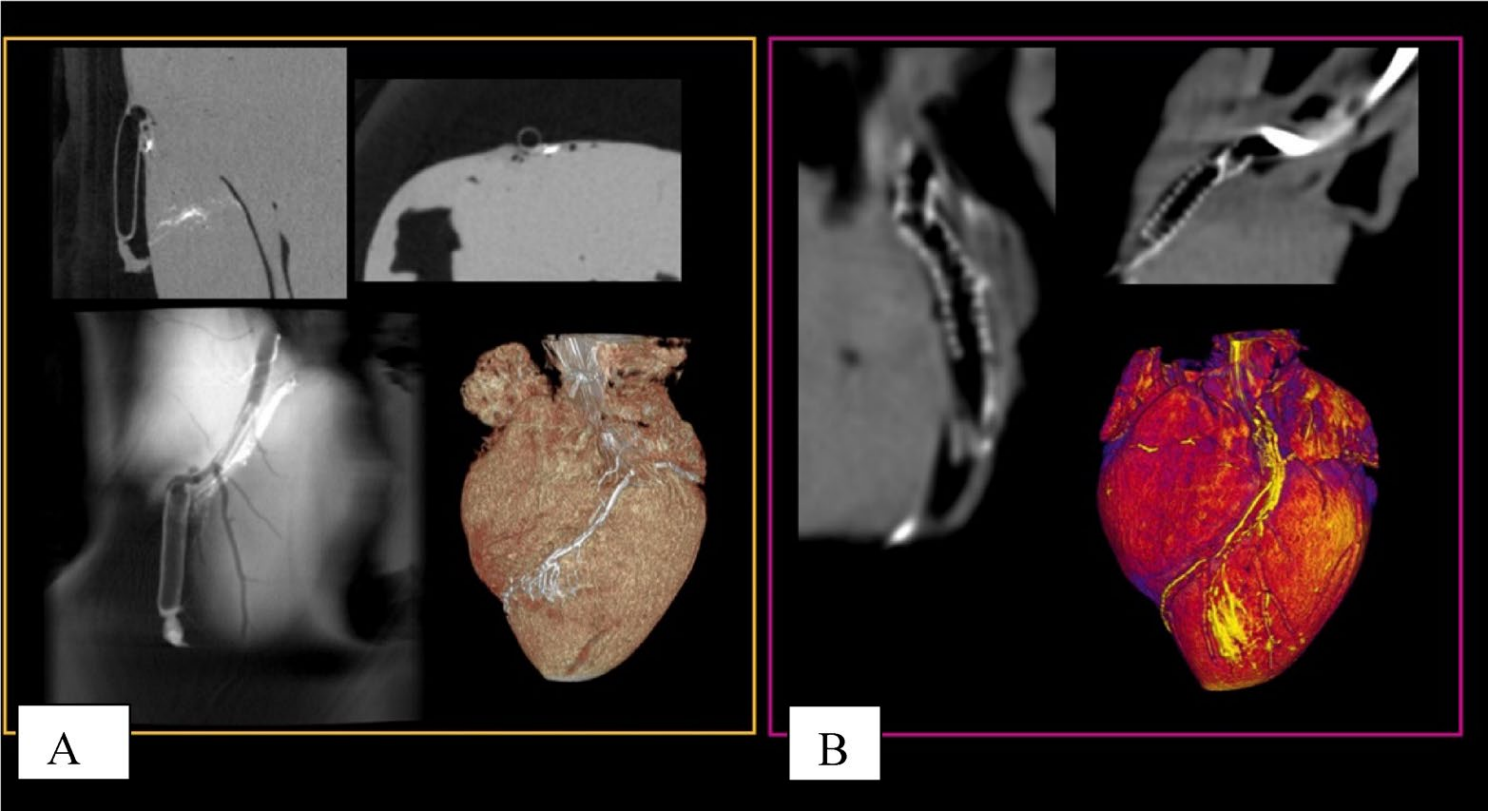
(**A**) Multi-dimensional display, maximum intensity projection (MIP), and volume rendering technique (VRT) of the inflated porcine heart after implementation of the AAP and a bypass). (**B**) demonstrates multi-dimensional views and VRT after placement of both, plaque and stent, inserted into the LAD.

Figure 5 shows the application of the AAP in the LAD in the imaging of a robot-assisted angiography system (Artis pheno^®^, Siemens Healthcare, Forchheim, Germany).

### Nuclear medicine

#### Visual analysis of cardiac lymphoscintigraphy and myocardial perfusion imaging

**Fig. 6:**
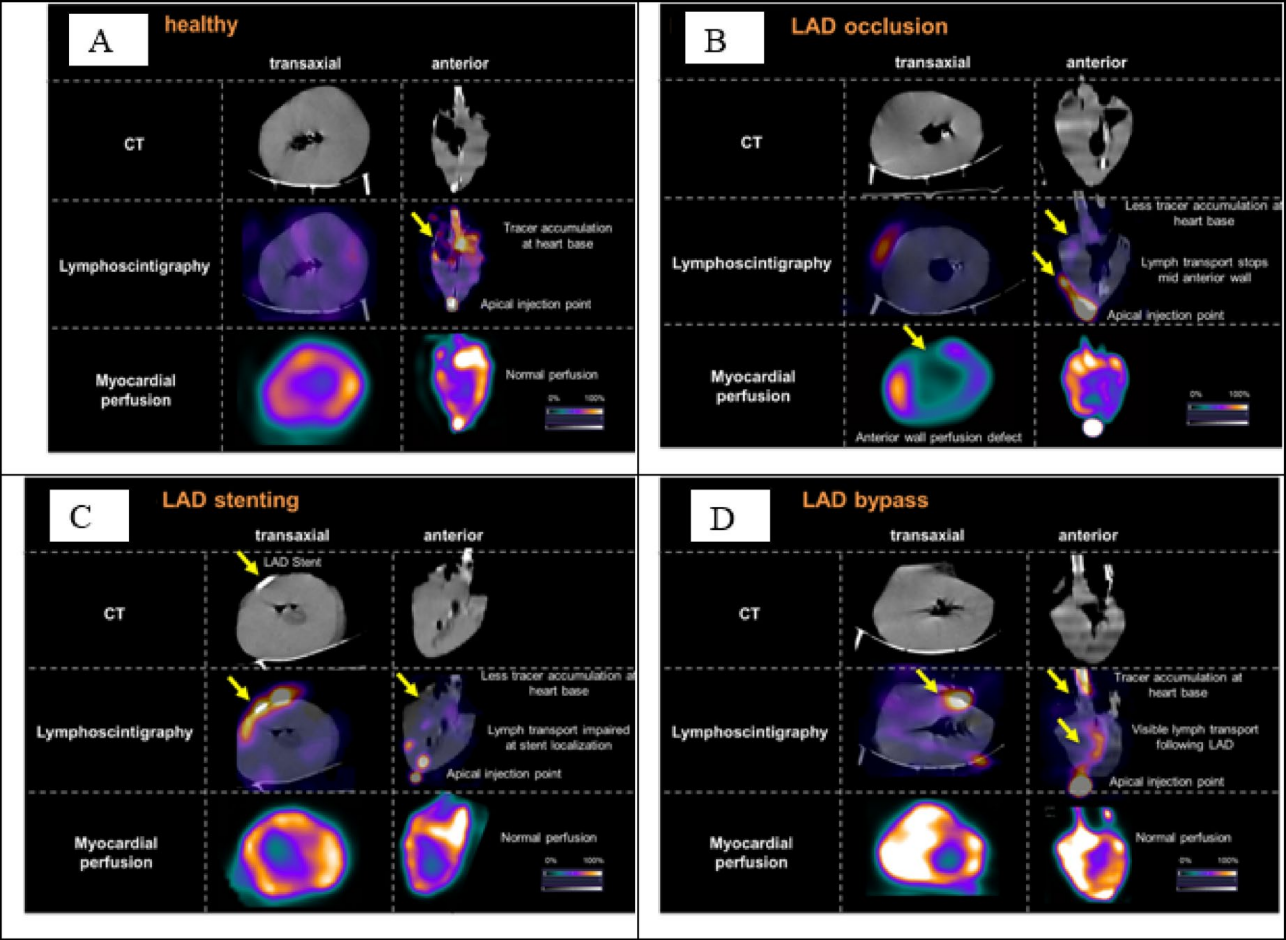
Nuclear cardiologic molecular imaging (lymphoscintigraphy and myocardial perfusion imaging) and CT scans of the in a representative explanted heart (**A**) and in an explanted heart with prior LAD occlusion (**B**), an explanted heart prior LAD stenting (**C**) or prior LAD bypass (**D**).

In healthy hearts without interventions (Fig. 6A, *n* = 3) lymphoscintigraphic images showed a high tracer accumulation at the heart base at the end of scan time. Apical tracer injection point was clearly visible as hotspot. MPI showed a homogeneous tracer distribution in the left ventricular myocardium without local perfusion defects. One heart was excluded from analysis due to intraventricular tracer injection.

In hearts with LAD occlusion (Fig. 6B, *n* = 2) lymphoscintigraphic images showed less tracer accumulation at the heart base when compared to healthy hearts. Apical tracer injection point was clearly visible as hotspot, from there lymph transport started following LAD and discontinued at mid anterior wall. Here, MPI showed a significant local perfusion defect of the anterior wall indicating myocardial infarction. Other myocardial areas showed homogeneous tracer distribution.

In both, lymphoscintigraphy and myocardial perfusion imaging, the apical tracer injection point is visible. While the healthy example shows a high tracer accumulation at the heart base with homogeneous left ventricular perfusion, the LAD occlusion example shows less tracer accumulation at the heart base and a stop of lymph transport in the mid anterior wall. Here, a matching perfusion defect is present.

In hearts with LAD stenting (Fig. 6C, *n* = 4) lymphoscintigraphic images showed less tracer accumulation at the heart base when compared to healthy hearts. Apical tracer injection point was clearly visible as hotspot, from there lymph transport started following LAD and got impaired at stent location in the mid anterior wall. Here, MPI showed no local perfusion defects, complete left ventricular myocardium presented homogeneous tracer distribution. One heart was excluded from analysis due to partial intraventricular tracer injection.

In hearts with bypass surgery (Fig. 6D, *n* = 4) lymphoscintigraphic images showed high rates of tracer accumulation at the heart base indicating a sufficient cardiac lymph transport. Apical tracer injection point was clearly visible as hotspot, from there lymph transport started following LAD and continuously reached the heart base. Here lymph transport did not discontinue in the mid anterior wall in comparison to hearts with LAD occlusion or stenting. Again, MPI showed no local perfusion defects. One heart was excluded from analysis due to intraventricular tracer injection.

Presence of regional differences in lymph transport was examined using volumes of interest (VOI) analysis and expressed as percentage of myocardial radioactive counts. VOIs refer to specific, predefined regions within an imaging dataset that are selected for detailed analysis. In the context of this study, VOIs were placed in the left ventricular myocardium to evaluate lymphatic transport and myocardial perfusion in different coronary territories, such as the LAD, LCX and right coronary artery.​ They were used to measure the mean radioactive counts in these regions, which reflect the tracer accumulation and lymphatic transport efficiency. ​ By comparing the counts across different coronary territories, the researchers could assess regional differences in lymphatic flow and identify areas of impaired transport. ​This analysis helps quantify the impact of interventions (e.g., stenting or bypass surgery) on lymphatic transport and provides insights into how these treatments affect the heart’s physiology.

**Fig. 7:**
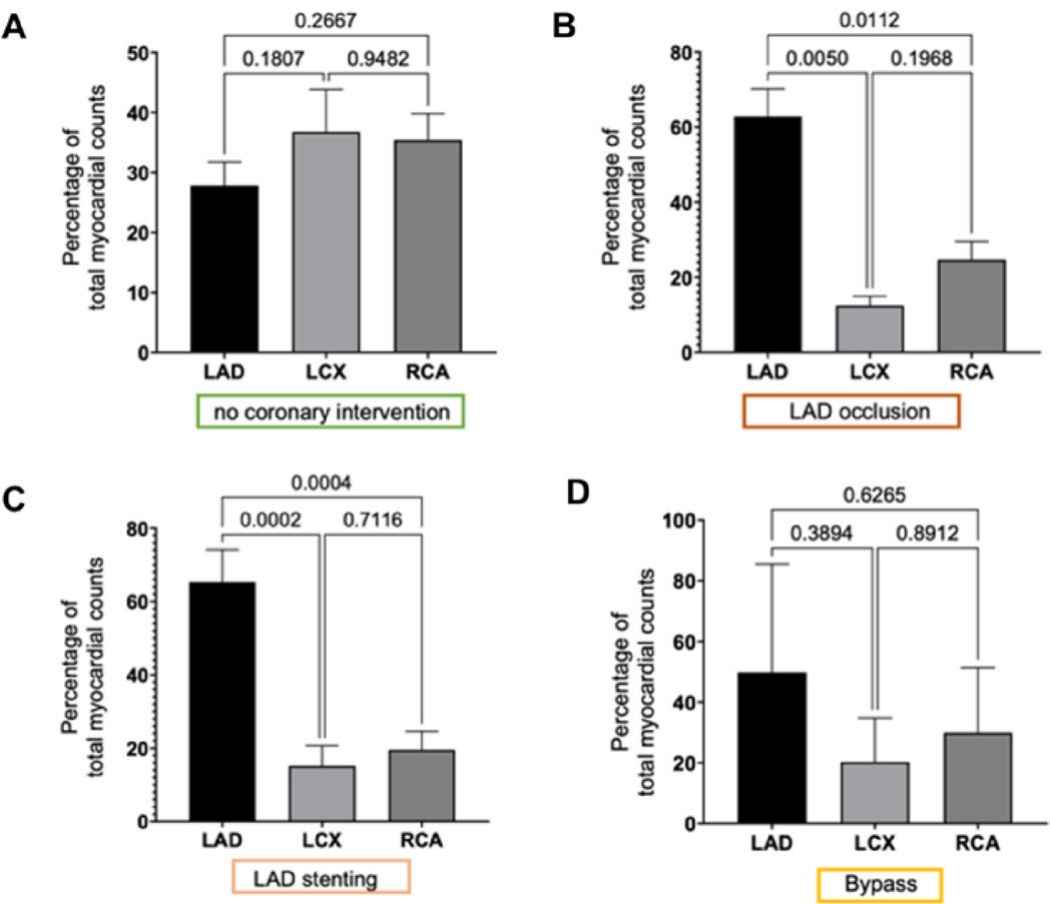
VOI analysis expressed as the percentage of total myocardial radioactive counts in the territories of the LAD, LCX, and RCA. The panels show data (**A**) from healthy hearts without coronary intervention (*n* = 4), (**B**) from the LAD occlusion group (*n* = 4), (**C**) from the LAD stenting group (*n* = 4), and (**D**) from the bypass surgery group (*n* = 4). The sample size (n) represents the number of biological replicates (individual animals). Bars represent mean ± SD. P-values from pairwise comparisons are indicated above each graph. In the LAD occlusion group (**B**), a significantly higher accumulation of radioactive counts was observed in the LAD territory compared to the LCX (*p* = 0.0050) and RCA (*p* = 0.0112). This effect was also seen in the LAD stenting group (**C**) (LAD vs. LCX, *p* = 0.0002; LAD vs. RCA, *p* = 0.0004). No significant differences between the territories were found in the no-intervention (A) or bypass (D) groups.

Regional semi-quantitative evaluation lymph transport in coronary territories Tracer retention was quantified by VOI analysis and expressed as the percentage of total myocardial radioactive counts in the territories of the left anterior descending artery (LAD), left circumflex artery (LCX), and right coronary artery (RCA) (Fig. 7).

In healthy hearts without coronary intervention, no statistically significant differences could be found between the coronary areas (Fig. 7A). It seems that along the non-affected 3 coronaries, the lymph is transported to the base in equal proportions. Both the infarction and stenting experiments showed a significantly higher percentage of myocardial radioactive counts proximal to the intervention in the LAD area (Fig. 7B and C). This likely corresponds to an impairment of lymph transport form apex to heart base in the LAD area or interruption of the regular transport. Of note, in the group with bypass surgery, this effect was not detectable.

Regional semiquantitative analysis of cardiac lymph transport.

### Histopathological examination

The Bypass group showed vital, non-stenotic, non-thrombotic and non-atherosclerotic anastomosis between the coronary artery and the bypass vessel. Typical signs former surgical procedure, such as anisotropic foreign material and hemorrhage could be demonstrated in every LAD section (from proximal, central and distal bypass). Signs of acute infarction, such as severe edema or myocardial necrosis were absent. The thorough assessment of peripheral nerves in epicardial tissue and the cardiac veins, smaller coronary branches and lymphatic vessels did not show any severe damage, drainage disorder or stenosis, respectively. Of note, initial signs of cardiac tissue damage caused by ischemia due to the AAP only become evident approximately four hours after infarction. Regarding the open anastomosis, total ischemia time of approx. 3.5 to 4 h (plaque implantation: < 20 s + explantation to OCS implantation time: max. 30 min = ~ 20–30 min, OCS time: 3 h), and further clinical testing, it is safe to assume that the bypass technique was successful. However, in our setting – initial – myocardial infarction and its extent can neither be fully confirmed nor ruled out histologically.

The Stent Group, under consideration of the before mentioned minimal duration after an ischemic event, also showed no signs of avital cardiomyocytes. All coronary arteries in the SG were non-stenotic and showed no signs of endothelial injury or thrombosis.

The AAP was likely dissolved by fixation and staining techniques and therefore could not be reliably demonstrated by conventional histopathology.

**Fig. 8:**
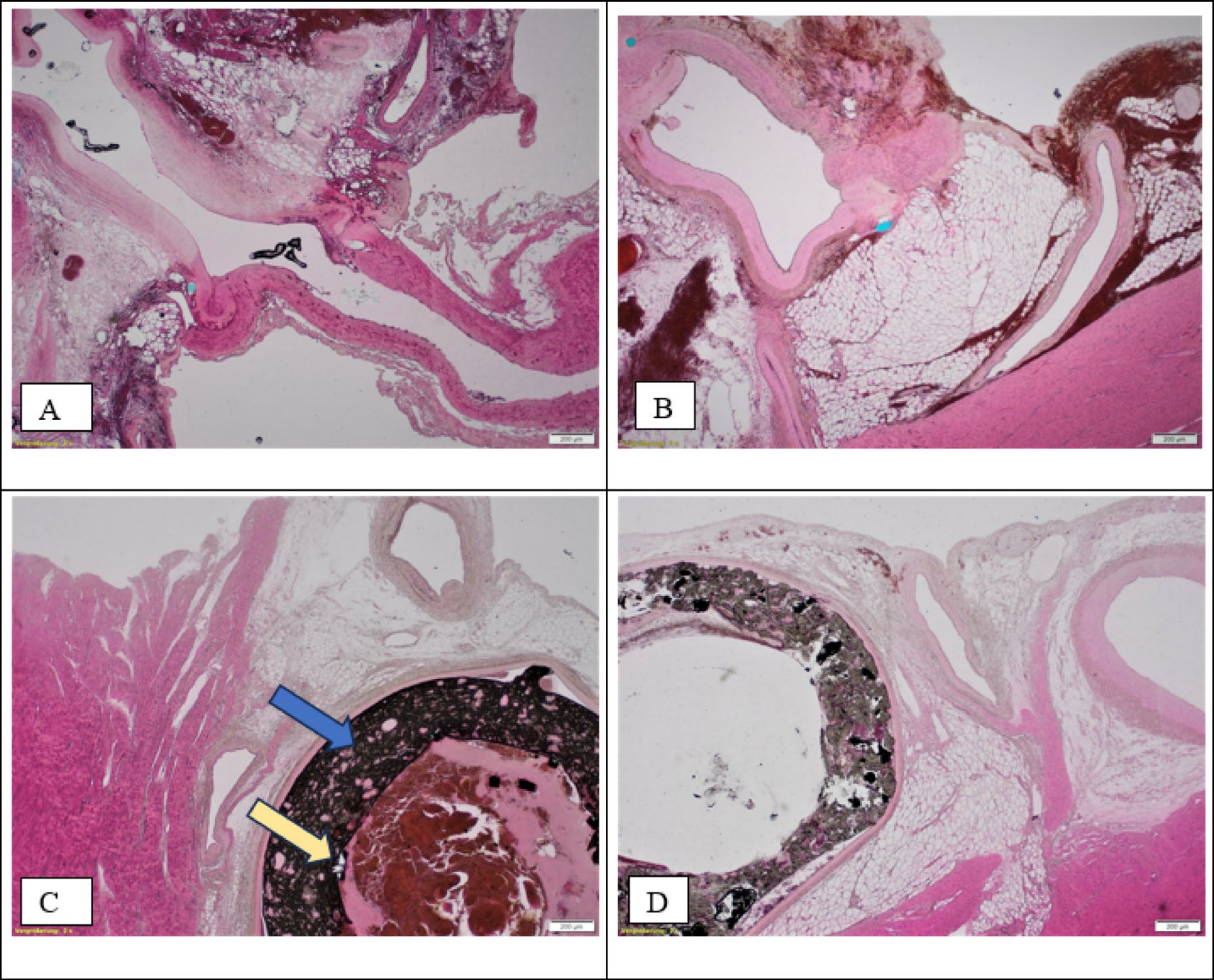
(**A**,**B**) (Bypass Group, Hematoxylin/Eosin-Elastica (HE-Elastica), 20-fold magnification). (**C**,**D**) (Stent Group, HE-Elastica, 20-fold magnification); Blue arrow: AAP, yellow arrow: Stent fragments; Scale bars = 200 μm.

Figure 8A,B show open and vital anastomosis between coronary artery and bypass vessel. Hemorrhages and foreign material due to surgery. No signs of infarction could be demonstrated (vital cardiomyocytes). Considering that initial signs of cardiac tissue damage caused by ischemia due to the artificial atherosclerotic plaque (AAP**) only becomes evident approximately four hours after the intervention. No severe injury of peripheral nerves, or drainage disorder of cardiac veins and lymphatic vessels could be demonstrated. Figure 8.C and 8.D (Stent Group, HE-Elastica, 20-fold magnification) show the coronary artery lumina with greyish stent material (Fig. 8C and intraluminal cruor postmortem clot, next to vital myocard. No residual particles of the AAP were shown.

In summary, artificial stenosis was absent after bypass surgery as well as intervention (see figures).

## Discussion

This newly developed animal model evaluated the comparative effects of PCI with stenting versus CABG in a porcine model of coronary heart disease using an AAP. While the current results provide significant proof-of-concept for the differential impact of CABG and PCI on lymphatic flow, the small sample size (*n* = 4 per group) limits the generalizability of the statistical findings. These results should be considered preliminary and serve as a foundation for future high-powered confirmatory studies. This research was intended to establish the feasibility of the newly developed AAP model and the ex-vivo OCS imaging protocol. The findings demonstrate that CABG provides superior outcomes in terms of both myocardial perfusion and lymphatic drainage. Hearts treated with CABG exhibited preserved or restored lymphatic transport, with no significant regional differences in tracer distribution. In contrast, PCI-treated hearts showed impaired lymphatic flow in the LAD territory, evidenced by increased tracer retention proximal to the intervention, although global myocardial perfusion remained intact. Collectively, these findings underscore the potential advantages of CABG over PCI in maintaining lymphatic function and myocardial tissue integrity in the setting of coronary artery disease.

Lymphoscintigraphy is a well-established imaging technique for visualization of lymph transport in vivo. Lymphoscintigraphies for detection of sentinel lymph nodes e.g. in breast cancer represent established routine practice and is recommended by international guidelines^44,45^. After intradermal application of 99mTc-labeled nanocolloid lymph vessels will take up the tracer and lymph transport to the next lymph node areas will be visualised by scintigraphy. Prior preclinical studies have demonstrated feasibility of cardiac lymphoscintigraphies in different animals (e.g. dogs, mice) intramyocardial tracer injection and heart in situ^46–49^. Our study is the first to perform cardiac lymphoscintigraphies of explanted pig hearts equipped with an OCS. Using a dedicated cardiac SPECT camera with high temporal and spatial resolution we were able to visualize cardiac lymph transport from apex to base following the coronary arteries. Apical tracer injection led to lymph transport predominately along the LAD, most likely based on individual coronary anatomy. We observed impaired lymph transport in hearts with coronary ligation and coronary stenting when compared to healthy hearts. Hearts with bypass surgery visually tended to have a better lymph transport from apex to heart base. Incidental intraventricular tracer injection occurred in 3 hearts resulting in exclusion from lymphoscintigraphic analysis. Myocardial perfusion was detected by MPI following lymphoscintigraphy on the same camera. Importantly, no significant regional perfusion defects were detected in healthy hearts, hearts with LAD stenting or bypass. Of note, the experimental setting of an explanted heart equipped with an OCS does not reflect a physiologic condition and may affects lymph flow and myocardial perfusion. However, the technical sensitivity of the lymphatic imaging was highlighted by the exclusion of three hearts due to suboptimal, intraventricular tracer injection. To ensure high-quality imaging and reproducibility, the depth of the apical intramyocardial injection is a critical variable that requires precise control to avoid ventricular cavity penetration. We propose that future applications of this protocol should incorporate real-time ultrasound guidance or standardized needle-stops to fix injection depth, thereby minimizing variability. Despite these challenges, the use of a dedicated cardiac SPECT camera successfully allowed for the first-ever visualization of lymph transport in an ex-vivo OCS setting, proving the fundamental viability of the method.

The histological examination revealed differences between the bypass and stent groups, particularly in terms of lymphatic flow and tissue integrity. ​ In the bypass group, the anastomosis between the coronary artery and the bypass vessel was vital, non-stenotic, and free of thrombosis or atherosclerosis. ​ Importantly, there were no signs of severe injury to peripheral nerves, drainage disorders in cardiac veins, or lymphatic vessels. ​ This suggests that bypass surgery preserves lymphatic flow and vascular integrity, potentially contributing to better long-term outcomes for the blood vessel and surrounding tissue.​.

In contrast, while the stent group also showed no signs of infarction or severe endothelial injury, the lymphatic flow was impaired in the LAD region proximal to the intervention. This disruption in lymphatic transport could lead to localized congestion, reduced drainage efficiency, and potentially contribute to chronic inflammation or tissue remodeling over time. ​ Although no immediate signs of tissue damage were observed, the impaired lymphatic flow may predispose the vessel to future complications, such as fibrosis or reduced vascular function. The bypass group demonstrated preserved lymphatic drainage, which is crucial for maintaining tissue homeostasis, reducing inflammation, and supporting vascular health. ​ This may lead to better long-term outcomes for the blood vessel and surrounding myocardium. ​ The stent group showed disrupted lymphatic transport, which could result in localized congestion and hinder the removal of inflammatory mediators. ​ Over time, this may negatively impact the vessel’s health, leading to chronic inflammation, tissue remodeling, or reduced perfusion efficiency. ​ The findings suggest that CABG may offer superior outcomes in terms of preserving lymphatic flow and vascular integrity compared to PCI. ​ While PCI is less invasive and has shorter recovery times, the potential for impaired lymphatic drainage and its long-term consequences should be considered, especially in patients with complex coronary artery disease. ​ Further studies are needed to explore the chronic effects of these interventions on lymphatic and vascular health.

The current experimental design focused exclusively on the acute physiological response within a narrow timeframe of approximately four hours post-intervention. While this allowed for a precise assessment of immediate mechanical and functional disruptions to the lymphatic system, it leaves critical long-term clinical concerns unaddressed. Specifically, processes such as chronic lymphatic remodeling, in-stent restenosis in the PCI group, and long-term graft patency in the CABG group could not be evaluated. Our findings of impaired lymphatic transport following stenting suggest a potential predisposition to chronic inflammation or fibrosis, yet these hypotheses require validation through chronic animal models. Future studies with extended follow-up periods are essential to bridge the gap between acute mechanical obstruction and long-term clinical outcomes.

Furthermore, the biological representativeness of the AAP must be considered. The AAP was engineered to mimic the clinical and physical dimensions of human stenotic lesions using a gelatine matrix mixed with cholesterol, phospholipids, and calcium components. While this composition successfully simulates the mechanical obstruction and radiological appearance of a 50% LAD stenosis, its biological behaviour may differ from human atherosclerotic plaques, which involve active inflammatory signalling and cellular heterogeneity. Specifically, the current AAP may not fully replicate the inflammatory signalling pathways, spontaneous plaque rupture, or the intricate cellular interactions of a chronic human lesion. The AAP results in varying ischaemic times also because of different LAD vessel diameters regarding to the pig’s heart size (even when choosing similarly aged pigs). Future iterations should explore the integration of bioactive signalling molecules or more complex cell-seeded scaffolds to enhance the biological relevance of the simulated plaque.

Finally, a primary limitation of the present study is the relatively small sample size, with only four animals (*n* = 4) assigned to each experimental group. While this cohort was sufficient to demonstrate statistically significant differences in lymphatic radioactive counts between the intervention groups, the overall statistical power remains limited. Individual anatomical variations and experimental conditions contributed to the variance observed in the nuclear medicine evaluation. To confirm the generalizability of our findings regarding the superiority of CABG in preserving lymphatic flow, future investigations with larger, high-powered cohorts are mandatory to account for individual anatomical and physiological variability.

## Conclusion

The AAP with clinical and physical similarities to human atherosclerotic plaques are most promising as a model to create a helpful device for future atherosclerotic infarction modeling. The application of the AAP into a blood vessel of interest provides a novel technique for future medical trials and could help to test whether the pressure exerted intraluminally of a PCI, or the creation of a bypass anastomosis leads to a reduced blood flow in the distal vasa vasorum, a disruption of the lymphatic drainage or damage to the innervation. Even though the currently accessible immunohistological markers for innervation/lymphatics did not work satisfactory, new and better ones should be tested, as soon they are available.

The study shows the effectiveness of the OCS for testing artificial plaque in a living, beating porcine heart, and the system demonstrated excellent functionality. The OCS allowed the heart to be preserved outside the body under near-physiological conditions, ensuring precise tracer application and stable circumstances. The almost complete maintenance of heart function throughout the experiment enabled a realistic simulation and analysis of the interactions between the plaque and heart tissue. These results highlight the potential of the OCS as a reliable model for cardiovascular studies, particularly for exploring new therapeutic approaches to coronary artery disease.

The infarction and stenting experiments revealed significantly higher mean counts in the LAD area proximal to the intervention, indicating “congestion” or disrupted transport. This effect was not observed in the bypass group.

The use of the AAP for the simulation of chronic heart disease has the potential to occlude a blood vessel without interfering with the entire organism experiencing atherosclerotic formations. It can be applied percutaneously like a stent and inserted into any blood vessel. This new established animal model can further be used evaluate the consequences of multiple atherosclerotic changes.

In summary, intravascular artificial atherosclerotic plaque testing holds great promise in revolutionizing our understanding of cardiovascular disease, fostering innovation in diagnostics and therapeutics, and ultimately improving patient outcomes. As researchers continue to push the boundaries of scientific knowledge in this field, the potential for transformative advancements in cardiovascular medicine becomes increasingly tangible.

## Data Availability

The datasets generated and/or analysed during the current study are available from the corresponding author on reasonable request.
